# The afterlife of a horizontally transferred gene: Expansion and functional diversification of a C1A peptidase in wild *Hordeum* species

**DOI:** 10.1002/tpg2.70242

**Published:** 2026-08-27

**Authors:** Václav Mahelka, Petra Caklová, Radim Čegan, Simón Villanueva‐Corrales, Jiřina Josefiová, Jana Kneřová, David Kopecký, Michaela Nagy‐Nejedlá, Marek Szecówka, Karol Krak

**Affiliations:** ^1^ Department of Evolutionary Plant Biology Institute of Botany, Czech Academy of Sciences Průhonice Czech Republic; ^2^ Molecular Biology and Bioinformatics Lab Institute of Botany, Czech Academy of Sciences Průhonice Czech Republic; ^3^ Centre of Plant Structural and Functional Genomics Institute of Experimental Botany, Czech Academy of Sciences Olomouc Czech Republic; ^4^ Department of Plant Developmental Genetics Institute of Biophysics, Czech Academy of Sciences Brno Czech Republic; ^5^ Faculty of Environmental Sciences Czech University of Life Sciences Prague Prague Czech Republic

## Abstract

Horizontal gene transfer (HGT) can accelerate plant adaptation, but the evolutionary fate of newly acquired nuclear genes is often unclear. Here, we analyzed a papain‐like cysteine peptidase horizontally transferred from Panicoideae to wild *Hordeum* (Poaceae). Using chromosome‐level assemblies of 21 diploid *Hordeum* species, we assessed presence/absence, copy‐number variation, coding diversity, selection, predicted protein functionality, and expression. To infer donor relationships, we screened 77 Panicoideae accessions (48 species, 20 genera) by polymerase chain reaction and sequenced positive amplicons with Nanopore. The transferred locus, likely acquired as part of a larger panicoid DNA segment, is retained across *Hordeum* sect. *Stenostachys* but shows strong post‐transfer diversification, with 1–8 copies per species, extensive transposable‐element insertions, and numerous coding‐sequence variants. Close homologs in Panicoideae were rare and restricted to *Panicum* and *Paspalum*; gene trees identified *Panicum bergii* as the closest sampled relative. Selection analyses indicated heterogeneous constraints and episodic positive selection in some *Hordeum* lineages. Only a minority of copies retained an intact catalytic triad, whereas most were predicted to be pseudopeptidases. Transcripts were detected across species and tissues. These results indicate that an HGT‐derived DNA block can persist, amplify, and diversify in recipient genomes, generating raw material for later functional evolution.

## Plain Language Summary

Genes are typically passed from parents to offspring, but occasionally, a gene can “jump” between plant species. This process, known as horizontal gene transfer, can accelerate evolution. We followed one transferred gene that once coded for a protease—an enzyme that breaks down proteins—in wild relatives of the barley genus *Hordeum* to see what happened after it arrived. Evidence suggests that it originated in a different group of grasses, with *Panicum bergii* identified as the likely source. This gene is widespread within the *Hordeum* section *Stenostachys*, but it is highly variable; species carry between one and eight divergent copies. While most copies no longer resemble active proteases, many are still active in several species and tissues. This demonstrates that a foreign gene can duplicate and diversify to become a regulated gene family, which may enable plants to explore new functions.

**Abbreviations**
aBSREL: adaptive branch‐site random effects likelihood
BH‐FDR: Benjamini–Hochberg false discovery rate
BLAST: Basic Local Alignment Search Tool
CDS: coding sequence
HGT: horizontal gene transfer
LRT: likelihood ratio test
MEME: mixed effects model of evolution
ML: maximum‑likelihood
MSA: multiple sequence alignment
NCBI: National Center for Biotechnology Information
ONT: Oxford Nanopore Technologies
PCR: polymerase chain reaction
PLCP: papain‐like cysteine endopeptidase
plDDT: predicted local distance difference test
SLAC: single‐likelihood ancestor counting
T‐DNA: transferred DNA
TE: transposable element
TPM: transcripts per million

## 1 INTRODUCTION

Horizontal gene transfer (HGT) is increasingly recognized as a potentially important evolutionary mechanism in plants (R. Chen et al., 2021; Mariault et al., 2025; Wickell & Li, 2020). Although its occurrence in higher plants has been recognized only relatively recently (Diao et al., 2006), its overall extent and evolutionary significance remain incompletely understood. The persistence of captured genes and their functions in plant genomes suggests that HGT can have adaptive value (R. Chen et al., 2021; Wickell & Li, 2020). For example, HGT has played crucial roles in the evolution of ferns (Li et al., 2014), the emergence of parasitism in plants (Yang et al., 2016), and the C_4_ metabolic pathway in grasses (Christin et al., 2012). In such cases, HGT acts as an “evolutionary shortcut” that accelerates adaptation (Phansopa et al., 2020; Wickell & Li, 2020).

Plant‑to‑plant transfers frequently involve mitochondrial genes and transposable elements (TEs) (Bock, 2010; Park et al., 2021), whereas plastid and nuclear gene transfers are rarer. While nuclear DNA transfers between vascular plants have been reported mainly in species growing in intimate association (e.g., host‐parasite; Kado & Innan, 2018; Yang et al., 2016; Yoshida et al., 2010), the body of cases illustrating the transfers between non‑intimately associated plants is increasing (e.g., Christin et al., 2012; Diao et al., 2006; Dunning et al., 2019; El Baidouri et al., 2014; Hibdige et al., 2021; Mahelka et al., 2017; Mariault et al., 2025; Roulin et al., 2008; Vallenback et al., 2008).

We previously documented extensive HGT of yet undetermined adaptive value (Mahelka et al., 2017, 2021). Over the past ∼5 million years, wild species of the grass genus *Hordeum* (Poaceae, Pooideae) acquired foreign DNA through multiple transfers from at least five genera of panicoid grasses (Poaceae, Panicoideae). Sixteen of the 21 currently recognized diploid *Hordeum* species (all species of section *Stenostachys*; for infrageneric classification, see Blattner, 2009) show evidence of HGT, with North and South American species containing the highest amount of foreign DNA. We infer that the oldest transfer involved *Panicum* and introduced a set of stress‑related protein‑coding genes into *Hordeum* genomes (Mahelka et al., 2021). One such gene, whose closest Basic Local Alignment Search Tool (BLAST) match in National Center for Biotechnology Information (NCBI) was an Ervatamin‑C‑like C1A peptidase (XP_025822421) from *Panicum hallii*, lacks a native copy in *Hordeum* and shows transcript expression in some species (Mahelka et al., 2021). It therefore represents a novel, presumably functional gene in the *Hordeum* gene pool that merits further investigation.

Papain‐like cystein peptidases (MEROPS classification C1A) are a major class of plant cysteine proteases involved in protein turnover and processing, with roles spanning development, senescence, immunity, and stress responses (Liu et al., 2018; Richau et al., 2012). They are synthesized as preproenzymes (inactive precursors carrying signal peptide and inhibitory prodomain) and are commonly targeted to proteolytically active compartments such as the vacuole and apoplast, consistent with central functions in intracellular remodeling and extracellular defense (Misas‐Villamil et al., 2016; Richau et al., 2012). In plants, members of this family have been linked to seed reserve mobilization, programmed cell death, senescence, and pathogen defense, indicating substantial functional specialization across tissues and biological contexts (Liu et al., 2018; Richau et al., 2012). Many C1A peptidases are also stress responsive, with several members induced by dehydration, salinity, and osmotic stress, suggesting roles in proteome reorganization and nutrient remobilization under adverse conditions (Liu et al., 2018). Because papain‐like peptidases can either enhance stress acclimation and defense or promote senescence and cell death, depending on the context, they may contribute to plant adaptation by regulating protein degradation under changing environmental conditions (Liu et al., 2018; Misas‐Villamil et al., 2016).

What remains unclear is the function, copy number, sequence variation, and evolution of the gene in *Hordeum*, as well as its donor lineage. Its presence in *Pan. hallii* genome suggested transfer from a *Panicum* species, yet its absence from other *Panicum* genomes (notably *Pan. miliaceum* and *Pan. virgatum*) and from a broad set of other grasses (64 genomes from NCBI with at least contig‑level assemblies screened) reported earlier (Mahelka et al., 2021) casted doubt on this hypothesis. Recently, annotated genomes of all diploid *Hordeum* species have become available as part of the *Hordeum* pangenome study (Feng et al., 2025). These genomes represent an unprecedented opportunity to find answers to the above‐mentioned questions related to C1A peptidase. Here, we aim to (1) quantify copy number and sequence diversity of the C1A peptidase locus in *Hordeum*; (2) map its distribution within Panicoideae by combining sequence database mining with targeted polymerase chain reaction (PCR) in selected accessions; (3) identify the closest Panicoideae relative (putative HGT donor) using phylogenetic analyses; (4) assess signatures of selection shaping sequence divergence; (5) predict molecular function from motifs and 3D models to classify C1A peptidase homologs as functional versus pseudopeptidases; (6) profile the gene expression across *Hordeum* homologs to test for regulatory subfunctionalization.

## 2 MATERIALS AND METHODS

### 2.1 Characterization and distribution of the C1A peptidase in *Hordeum*

To examine presence and copy‐number of the C1A gene in *Hordeum*, we used annotated chromosome‐level genome assemblies (Feng et al., 2025) including a single genotype representative of each diploid *Hordeum* species (Table S1). Gene sequences were identified with BLAST in Geneious R10 (Biomatters Ltd.) using the Ervatamin‐C‐like sequence from *Hordeum bogdanii* as a query (localized at positions 123,561–124,757 in BAC clone MT507784; Mahelka et al., 2021). The BLAST hits were intersected with genome annotations and gene sequences were extracted for subsequent analyses. Individual species of *Hordeum* have none, one, or multiple copies of the gene. Hereafter, we use the term homolog as a general designation for any copy of the gene, irrespective of its evolutionary path. Terms ortholog and paralog are used for gene copies evolved due to speciation and duplication, respectively, as usual. From the genome assemblies, we also extracted annotated TEs and recorded their classifications and positions relative to the C1A gene. Because the gene contained diverse TEs in most homologs across species, we focused on the coding region. The coding sequences (CDSs) were extracted, aligned with MAFFT v. 1.4.0 implemented in Geneious and translated for domain verification using InterPro database online (Blum et al., 2025). All sequences were identified as papain‑like cysteine peptidase C1A (see Results). Each homolog was assigned a unique ID indicating the species name, the C1A identifier, and a numeric suffix when multiple paralogs occurred within one accession. If a gene (homolog) contained multiple transcript isoforms with distinct CDSs, they were labeled with the suffix “CDS” followed by an isoform number.

### 2.2 Characterization and distribution of the C1A peptidase in Panicoideae

The presence of the C1A gene was previously confirmed in *Panicum hallii* var. *filipes* (Mahelka et al., 2021). The presence of the gene in the second genotype (var. *hallii*) published by Lovell et al. (2018) was assessed by BLAST. Gene sequences of *Panicum* and *Hordeum* were combined and used to generate a multiple sequence alignment (MSA) with MAFFT. These alignments were then used to design primers for PCR screening in Panicoideae. Three forward and one reverse primer targeting the most conserved region were designed: EVAC_83F (5′‐AGTTCACCACACGACCTC‐3′), EVAC_371F (5′‐CACTTGTGCCCGTCAAAAGG‐3′), EVAC_508F (5′‐TGCGTAGGTCCTGTCGAA‐3′), and EVAC_988R (5′‐GAGTCGGGATCGTAACCCAC‐3′). EVAC_988R and EVAC_508F primers correspond to the reverse complements of EVAC_122F and EVAC_710R primers, respectively, from a previous study (Mahelka et al., 2021). Species representing major Panicoideae lineages were PCR‐screened for the C1A peptidase using verified accessions, which had been confirmed phylogenetically via the chloroplast *ndh*F marker (Supporting Information File S1; Table S2). Verified accessions (77 accessions from 48 species and 20 genera) were then PCR‐screened using the three primer combinations: EVAC_83F/EVAC_988R, EVAC_371F/EVAC_988R, and EVAC_508F/EVAC_988R. PCRs were performed with COMBI PPP master mix (Top‐BIO, Vestec, CZ) and 200 nM primers under the following cycling: 95°C, 5 min; 35 cycles of 95°C, 30 s–58°C, 30 s–72°C, 1 min; final extension 72°C, 10 min; hold at 4°C. Reactions were run in triplicate and pooled to minimize amplification bias. For PCR screening, a sample was scored as positive when at least one reaction was positive. Because primers were designed in conserved regions of the alignment, but divergence outside the sampled taxonomic space may still occur, negative PCR results were treated as absence of detectable amplification under our assay rather than definitive genomic absence.

Positive amplicons were sequenced using Oxford Nanopore Technologies (ONT). Libraries were prepared with the Rapid Barcoding Kit 96 and sequenced on an R10.4.1 PromethION flow cell (PromethION P2 Solo). Raw data were basecalled and demultiplexed with Dorado (v0.9.1; model sup v5.0.0). Reads from each sample were mapped to the reference corresponding to the targeted amplicon (*Pan. hallii*, XM_025966636.1) using minimap2 (v2.28‑r1209; Li, 2018). Unmapped reads were discarded. Variant calling was performed with Clair3 (Zheng et al., 2022) using the ONT‑appropriate model; heterozygous variants were phased with WhatsHap (v2.8; Martin et al., 2016). When variants were detected, phase information split reads into two haplotype sets; otherwise, a consensus sequence was generated with Kindel (v1.2.1; Constantinides & Robertson, 2017). The sets were processed iteratively (variant calling and phasing) until no further evidence of heterozygosity remained, and a final consensus was produced. This approach ensures detection of all haplotypes at the amplified locus, provided that the variant caller (Clair3) and phaser (WhatsHap) correctly identify the relevant variants. Any variation not detected within a given haplotype subset is collapsed into the corresponding consensus sequence. This method can resolve haplotypes differing by as few as two single‐nucleotide polymorphisms (SNPs). ONT‐derived sequences from PCR‐positive Panicoideae accessions were therefore treated as haplotypes (amplicon sequence variants) for phylogenetic analysis and were not interpreted as direct estimates of locus number. The resulting sequences were assigned IDs composed of the species name, a numeric accession identifier (if applicable), the C1A gene identifier, and a suffix ranging from a to c for multiple paralogs within an accession. The sequences were aligned with *Hordeum* and *Pan. hallii* homologs to define the reading frame, translated, and screened for internal stop codons; sequences containing internal stops were excluded from downstream analyses. The remaining sequences were translated and scanned for domain architecture as described above.

The distribution of species carrying the C1A peptidase was interpreted and visualized in a Panicoideae phylogenetic framework. We used the chloroplast *ndh*F dataset used previously for the phylogenetic verification; only the MSA was pruned to remove unverified samples and duplicates. In total, the MSA contained 298 samples (Table S3) and 795 sites. The maximum‑likelihood (ML) tree was built as described in Supporting Information File S1. Positively and negatively tested samples were mapped onto the tree using the ape (Paradis & Schliep, 2019) and phytools (Revell, 2024) packages in R 4.5.0 (R Core Team, 2025).

### 2.3 Phylogenetic analyses of the C1A peptidase

The goal was to investigate relationships among homologs in different *Hordeum* species and to identify their origin by placing them within Panicoideae. We conducted three analyses:

1. Position of *Hordeum* homologs within Panicoideae (protein level). To obtain a broader phylogenetic context, sequences of *Hordeum* and Panicoideae (*Panicum* and *Paspalum*, retrieved from the genome assembly of *Pan. hallii* and PCR screening) were analyzed together with additional Panicoideae proteins. Retrieval of the additional proteins proceeded as follows: (i) a translation of a representative *Hordeum* copy (*H_bogdanii*_C1A_6_CDS) served as a BLASTp query against the NCBI non‐redundant protein database (nr) and against Phytozome; hits with ≥40% coverage and ≥40% identity were retained. Duplicates were removed. When multiple sequences per species were retrieved, a few representatives were selected based on UPGMA in Geneious. (ii) Reviewed SwissProt records were retrieved by the query “peptidase C1A” filtered to Panicoideae (taxonomy_id: 147369). In this way, we obtained another 12 sequences. Two paralogs of a *Zea mays* accession (Q10716 and Q10717) retrieved from SwissProt were discarded because it was not possible to align them to other sequences. Sequences were assigned IDs where the number after the species name indicates the individual (if multiple) and the letter indicates the paralog (if multiple per individual). All sequences were scanned in InterPro to confirm functional classification and domain architecture. An MSA of 71 sequences and 516 sites was produced with MAFFT and manually refined (Supporting Information File S2). An ML analysis was run on the IQ‑TREE web server (Trifinopoulos et al., 2016) with automatic model selection, FreeRate heterogeneity, and 1000 ultrafast bootstrap replicates. Since no confidently alignable external outgroup was available, the resulting tree was midpoint‑rooted.
2. Position of *Hordeum* homologs within *Panicum* and *Paspalum* (protein level). Guided by the ML tree from step (1), we performed a focused analysis with limited taxon sampling. The dataset included *Hordeum*, *Pan. hallii*, and PCR‑amplified sequences from Panicoideae (*Panicum* and *Paspalum*). The MSA comprised 61 sequences and 466 amino acids (Supporting Information File S3). An ML tree was constructed as in (1), using *Pan. hallii* (*Panicum*_*hallii*_FIL_C1A_chr06) as the outgroup.
3. Position of *Hordeum* homologs within *Panicum* and *Paspalum* and inference of origin (nucleotide level). To increase resolution among closely related taxa and identify a potential donor of the gene in *Hordeum*, we built an ML tree using the complete CDS. Taxon sampling and phylogenetic settings matched step (2). A codon‑based alignment (1398 sites) was generated and edited in MAFFT implemented in Geneious (Supporting Information File S4).

### 2.4 Selection tests

To interpret substitution patterns in *Hordeum* C1A homologs and relate them to functional divergence, we analyzed a codon‐based nucleotide alignment and its ML phylogeny. We excluded from the alignment *H_cordobense*_C1A due to insufficient overlap with *Panicum_bergii*_C1A_1 (used as the closest non‐*Hordeum* comparator in relative‐rate [RR] tests), trimmed 83 upstream codons present only in *H_bogdanii*_C1A_4, and removed 138 nt at the 3′ end of *H_chilense*_C1A_1 due to a frameshift‐inducing deletion, yielding a final matrix of 60 sequences and 383 codons.

Prior to analyses, we assigned IDs to all internal branches to ensure consistent reporting. In the ML tree with transformed branches (option “equal”), branches were numbered sequentially by left‑to‑right, parent‑to‑child (preorder) traversal, starting at the root with the leftmost descendant. Branch IDs correspond to node IDs; for example, branch ID4 connects nodes ID3 and ID4 (Table S4; Figure S1). All results are reported using this universal branch list.

As a first‐pass screen for lineage‐specific rate acceleration, we applied Tajima's RR test (Tajima, 1993) in MEGA11 (Tamura et al., 2021) in a pairwise triplet setting on codon‐aligned CDS (A = a *Hordeum* homolog; B = *Pan. bergii_*C1A_1; O = *Pan. hallii* outgroup), running the test on three partitions (all codon positions; first + second; third) and controlling multiple testing across all triplets/partitions using Benjamini–Hochberg false discovery rate (BH‐FDR; *q* < 0.05). To assess whether the lack of signal at third codon positions could be explained purely by lower information content, we performed a post hoc power check. For each triplet with significant acceleration on first + second positions (*n* = 12), we computed r on first + second positions and predicted the expected *χ* ^2^ for the third‑position partition at its observed number of informative sites m_3_ as *χ* ^2^_pred_ = *r* ^2^·m_3_ (df = 1). We then counted how many triplets would be significant at *α* = 0.05 under this prediction and evaluated whether this fraction exceeded 0.5 using an exact two‑sided binomial test in R.

To move from “rate shifts” to explicit selection models, we then tested for changes in selection intensity on two prespecified foreground clades (clades 13 and 38, defined on the ML tree) using RELAX (Wertheim et al., 2015) on Datamonkey (Weaver et al., 2018), contrasting each foreground set against the remaining branches and assessing significance by a likelihood ratio test (LRT) of *K* ≠ 1 (with BH‐FDR adjustment across the two planned RELAX tests). Because RELAX informs on the *intensity* of selection but does not directly test for *ω* > 1, we next used BUSTED (Murrell et al., 2015) on Datamonkey as a gene‐wide test for episodic diversifying selection on each focal clade (foreground vs. background; default HyPhy settings including synonymous‐rate variation), with significance assessed by an LRT. To localize signals within significant foreground sets, we applied an adaptive branch‐site random effects likelihood (aBSREL) model (Smith et al., 2015) to identify specific branches showing evidence of episodic positive selection (*ω* > 1 at a subset of sites), using HyPhy's built‐in correction for multiple testing across the tested branches. In parallel, we used a mixed effects model of evolution (MEME) test (Murrell et al., 2012) to identify individual codon sites subject to episodic diversifying selection (site‐level LRT; BH‐FDR across sites; reporting threshold *p* ≤ 0.10), and we flagged branches contributing to each significant site from branch‐wise posterior support in the JavaScript Object Notation output. Finally, to map selected sites to concrete nucleotide and amino acid changes, we used single‐likelihood ancestor counting (SLAC) on Datamonkey/HyPhy to reconstruct ancestral codon states and infer substitutions along branches (Kosakovsky Pond & Frost, 2005), intersecting these substitutions with MEME‐supported branch–site combinations; physicochemical impact of nonsynonymous changes was summarized using Grantham distances (Grantham, 1974).

### 2.5 The inference of functionality in *Hordeum* C1A homologs

#### 2.5.1 Sequence‐based inference of functionality

Given that catalytic activity in papain‐like cysteine peptidases is determined by the mature domain, while the prodomain mainly acts as an autoinhibitory region requiring proteolytic removal for activation, we restricted our functional analysis to the catalytic competence of the mature domain. The processing and regulatory contribution of the prodomain were therefore not examined here. To assess whether *Hordeum* homologs retain catalytic protease activity, we classified C1A paralogs as likely functional when the catalytic CGxC block was present (the second Cys taken as the nucleophile), together with the canonical His and Asn of the Cys–His–Asn triad and a nearby oxyanion hole Gln (papain Gln19, i.e., 6 aa before the second Cys) in the expected N‑terminal vicinity (Ménard et al., 1991; Storer & Ménard, 1994). By contrast, sequences with CGSS (Cys→Ser at the catalytic position) or with His/Asn missing were labeled likely nonfunctional, consistent with the routine use of C25S/A “dead” mutants in cathepsins and their inactive crystal structures (Adams‑Cioaba et al., 2011; Turkenburg et al., 2002). We labeled as uncertain the atypical CG‑window variants (e.g., CSSC) that retain His/Asn and the Q‑oxyanion signature but deviate from CGxC. Prodomain motifs ERFNIN and GNFD (I29) were recorded but not used for catalytic calling, as they primarily govern zymogen inhibition/activation rather than catalysis per se (Aich & Biswas, 2018; Pandey et al., 2009). Finally, we note that MEROPS defines non‑peptidase homologs by replacement of catalytic residues, supporting our “nonfunctional” category when triad/oxyanion elements are lost (Rawlings et al., 2018).

#### 2.5.2 In silico predictions of the structure of the protein

In a subset of *Hordeum* C1A homologs, representing the likely functional, non‐functional, and catalytically uncertain proteins, we predicted protein structures using AlphaFold3 (Abramson et al., 2024) via the AlphaFold3 server. We used only the mature protein region (peptidase) for model predictions. We tested whether the proteins can fold into a 3D structure with a high predicted local distance difference test (plDDT) value (Jumper et al., 2021) and, assuming that the “likely functional” proteins have retained their catalytic activity and work properly, whether the active‐site geometry differs among the proteins of the three functional categories (uncertain vs. potentially functional, potentially non‐functional vs. potentially functional).

Visualization and analyses of the predicted structures were performed using UCSF ChimeraX version 1.11 (Meng et al., 2023). Local plDDT values were read from the plot of each 3D model at positions Gln(Ser)24, Cys/Ser30, His171(172), and Asn187(188). Active‐site geometry was summarized by three inter‐residue distances measured on best‐ranked AlphaFold/ColabFold models without manual side‐chain adjustment: OE1[Gln(Ser)24]–SG(Cys/Ser30), SG(Cys/Ser30)–ND1[(His171(172)], and ND1[(His171(172)]–OD1[(Asn187(188)]. To test the null hypothesis that geometry does not differ among functional, non‐functional, and uncertain classes, we applied the Kruskal–Wallis test separately to each distance (non‐parametric, three groups). When the omnibus test indicated a difference, we performed post hoc pairwise Mann–Whitney U comparisons with Benjamini–Hochberg correction for multiple testing within each distance. We report medians and consider *α* = 0.05 after correction.

#### 2.5.3 RNA expression of C1A peptidase in *Hordeum*

Expression of C1A homologs was evaluated using RNA‐seq data from the *Hordeum* pangenome study (Feng et al., 2025). A species was considered to express the gene if at least one homolog showed a defined expression level (see below) in at least one tissue. Expression was further examined by inferred functional class and across seven tissues in *Hordeum roshevitzii*. RNA‐seq data encompassed primarily leaf and root tissues, with inflorescences where available; 14‐day and 8‐day shoot samples from *H. bogdanii* and *Hordeum erectifolium* were treated as leaf tissue. Reads were quality‐filtered (Q30), and sequencing adapters were removed using Trimmomatic v0.32 (Bolger et al., 2014). Cleaned reads were mapped to the corresponding reference genomes with STAR v2.7.7a (Dobin et al., 2013) with default parameters and quantified using rsem v1.3.3 (Li & Dewey, 2011). Counts were converted to TPM (transcripts per million) using edgeR v4.2.1 (McCarthy et al., 2012). A gene was considered to be expressed if the TPM value was ≥0.5 in any tissue, following Expression Atlas recommendations (https://www.ebi.ac.uk/gxa/home). In addition, for visualization of expression levels across species, tissues, and homologs, TPM values were transformed to log_2_(TPM + 1), which reduced the skewness of the data and improved the display of both lowly and highly expressed transcripts. Because the RNA‐seq datasets were not generated under standardized growth conditions across species, interspecific differences in expression should be interpreted cautiously.

## 3 RESULTS

### 3.1 C1A is present in all but one *Hordeum* species, and its copy number varies

The gene was present in all species of sect. *Stenostachys* except *Hordeum brevisubulatum*, and individual species harbored between one and eight homologs (Table S5). A single homolog was found in *H. californicum*, *H. erectifolium*, *H. euclaston*, *H. flexuosum*, *H. pusillum*, *H. cordobense*, *H. intercedens*, and *H. muticum*. Length of the gene varied from 327 to 13885 nt, mostly due to the insertions of TEs. Most homologs contained TEs in various positions in their sequences, and only eight homologs (*H_bogdanii*_C1A_1, *H_chilense*_C1A_1 and 3, *H_cordobense*_C1A_1, *H_pubiflorum*_C1A_5, *H_roshevitzii*_C1A_3 and 4, and *H_stenostachys*_C1A_2) did not contain any TEs (Table S5). Both retrotransposons (LTR/Gypsy, LTR/Copia) and DNA transposons (CACTA, Mutator, hAT‐TIR, Tc1/Mariner) were identified. Most insertions were intronic, whereas a smaller number occurred in untranslated regions (5′ UTR and 3 ’UTR). In a single case, a Gypsy LTR overlapped the CDS in the opposite orientation relative to the gene (*H_bogdanii*_C1A_4) (Table S5). In total, there were 41 CDS variants (isoforms) in *Hordeum* species, whose lengths varied from 270 to 1275 nt. The codon‐based alignment of the CDS from the *Hordeum* species was straightforward (Supporting Information File S4). However, only the homolog *H_bogdanii*_C1A_4 contained 248 additional nucleotides at the 5′ end of the gene.

Using the InterPro database scan, we confirmed the membership of the proteins to papain‐like cystein peptidase C1A (InterPro IPR000668) in all *Hordeum* homologs except the truncated sequence of *H. cordobense*, whose insufficient length made identification impossible (Table S6). In other active sites and signal regions, the significant hits varied among the homologs due to the sequence variation.

### 3.2 C1A is patchily distributed in Panicoideae, restricted to *Panicum* and *Paspalum*

Two copies of the gene were retrieved from each of the two genomes of *Panicum hallii* (Table S7). One resided on chromosome 2 and the other on chromosome 6. The gene lengths were 711 and 1175 nt in var. *filipes* and 1162 and 2543 nt in var. *hallii*. CDS varied between 711 and 1059 nt in var. *filipes* and 981 and 1059 nt in var. *hallii*.

Among the PCR‐screened accessions, positive samples were restricted to species from the genera *Panicum* and *Paspalum* and included *Pan. barbipulvinatum*, *Pan. bergii*, *Pan. capillare*, *Pan. hallii*, *Pan. sumatrense*, *Pas. paucifolium*, and *Pas. scrobiculatum* (Table S2). Sequencing of the positive amplicons from 15 accessions of the seven species yielded a mixture of putative functional copies and pseudogenes, as determined based on internal stop codons. In all accessions except *Pan. hallii*_1, at least one putatively functional copy was detected (Table S8). The *Pan. hallii* genotypes used for PCR screening were different from the ones used by Lovell et al. (2018) for whole‐genome sequencing (Table S7), indicating interspecific variability. Likewise in *Hordeum*, all functional copies belonged to papain‐like cystein peptidases C1A (InterPro IPR000668) (Table S6).

**FIGURE 1:**
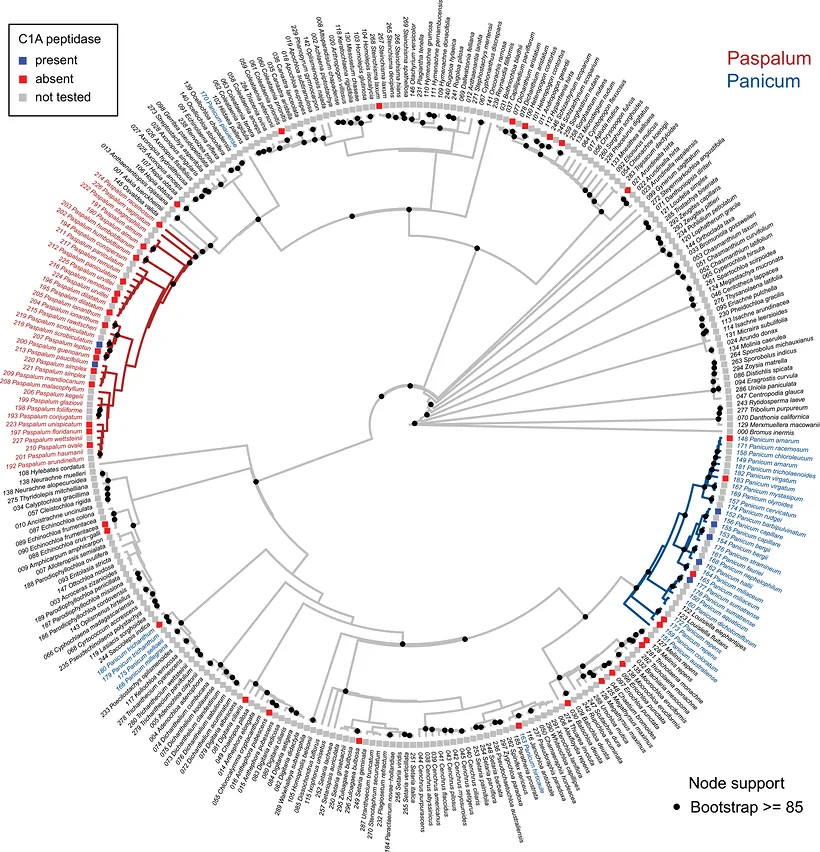
Distribution of the C1A peptidase within Panicoideae, inferred from polymerase chain reaction screening and mapped onto an *ndh*F chloroplast maximum‐likelihood (ML) tree.

**FIGURE 2:**
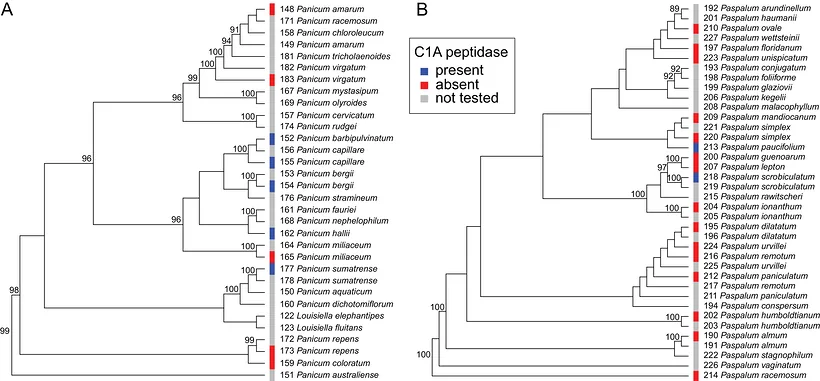
Distribution of the C1A peptidase within the genera *Panicum* (A) and *Paspalum* (B), inferred from polymerase chain reaction screening and mapped onto an *ndh*F chloroplast maximum‐likelihood (ML) tree.

The occurrence of the C1A peptidase was projected onto the chloroplast *ndh*F tree of Panicoideae (Figure 1). The gene is polyphyletic in the chloroplast tree, scattered among the genera *Panicum* and *Paspalum*. Neither within the two clades is the gene monophyletic (Figure 2). The scattered distribution of PCR‐positive accessions on the chloroplast *ndh*F tree should be interpreted cautiously, because chloroplast phylogeny may not fully track the history of the nuclear C1A locus (see Discussion). Thus, the plastid framework is used here to visualize distribution, whereas inference of closest relatives and donor context relies primarily on nuclear C1A gene trees.

### 3.3 *Panicum bergii* is the likely donor of C1A in *Hordeum* species

To place *Hordeum* C1A peptidase sequences within Panicoideae, we constructed an ML tree using complete protein sequences. The gene tree is divided into two major clades. *Hordeum* homologs formed a well‐supported clade together with *Pan. hallii* and the PCR‐amplified samples of *Panicum* and *Paspalum* (clade A, Figure S2). Within this clade, *Hordeum* homologs were sister to *Panicum bergii*. The rest of the species, that is, *Setaria*, *Urochloa*, and *Pan. virgatum*, formed a separate clade (B). In *Pan. virgatum* and *Urochloa decumbens*, the C1A was not monophyletic. We retrieved three sequences for both species. While in *Pan. virgatum* they came from three different genotypes (accessions), in the case of *U. decumbens* they represent paralogs from a single accession.

Next, we performed ML analysis of protein sequences and taxon sampling limited to A‐clade species from previous step. In this tree, again, *Hordeum* homologs formed a clade with *Pan. bergii* (Figure S3). *Hordeum* further formed several subclades, reflecting divergence in protein sequences. The sequence divergence reflects both species‐level diversification (speciation) as well as intragenomic duplication (paralogy).

**FIGURE 3:**
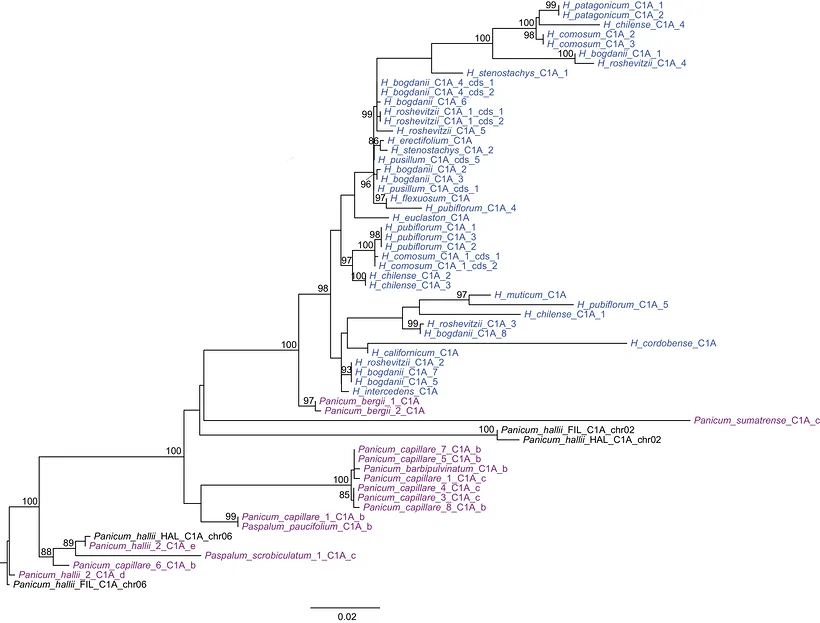
Maximum‐likelihood phylogeny of C1A peptidase homologs from *Hordeum* and their placement among closely related Panicoideae, inferred from nucleotide sequences of the coding region (coding sequence [CDS]). Blue color: *Hordeum* homologs; magenta: polymerase chain reaction‐amplified sequences of Panicoideae obtained in this study. Bootstrap support values <85% are not shown.

ML tree based on nucleotide data showed very similar topology to the ML tree based on protein sequences (Figure 3). The only marked difference is the placement of *Pan. sumatrense* and, within *Hordeum*, *H. cordobense*. The nucleotide tree places *H_cordobense* apart from *H_chilense*_C1A_1 in the nucleotide tree, implying that the protein‐based clade more likely reflected a data‐deficiency artifact (a larger number of informative sites in nucleotide alignment) instead of genuine relatedness.

In both protein and nucleotide trees, *Pan. bergii* is the closest relative to the *Hordeum* homologs. Therefore, based on the taxon sampling used here, *Pan. bergii* is the likely donor of the C1A gene in wild *Hordeum* species.

### 3.4 Episodic positive selection is the driver of sequence divergence

**FIGURE 4:**
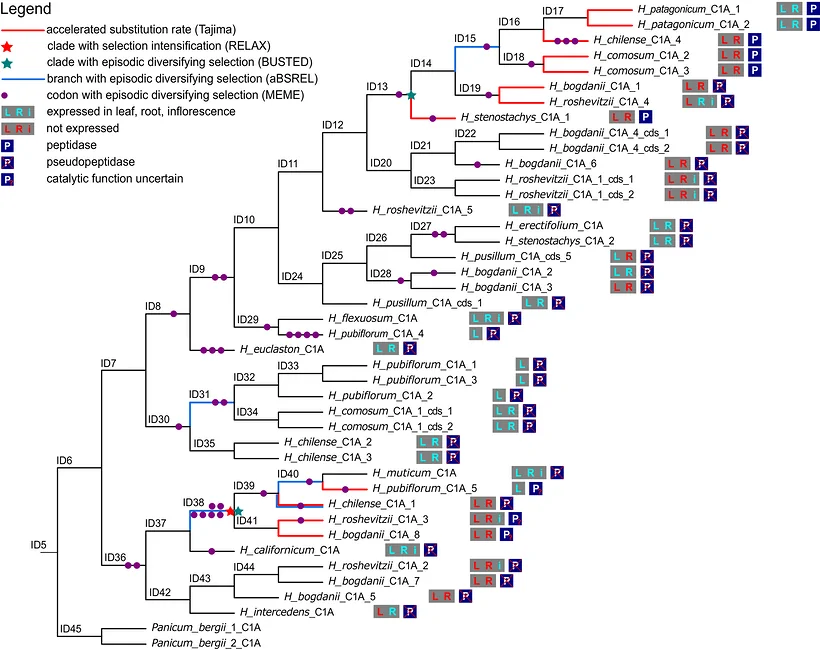
Summary of selection and functional analyses of the C1A peptidase in *Hordeum*. The C1A gene tree (nucleotide coding sequence [CDS]‐based maximum‑likelihood [ML] tree with transformed branch lengths) is annotated with results of tests for accelerated evolution (Tajima's relative‐rate test), changes in selection intensity (RELAX), gene‐wide episodic diversifying selection (BUSTED), adaptive branch‐specific episodic diversifying selection (aBSREL), and codon sites under episodic diversifying selection (mixed effects model of evolution [MEME]). Expression is based on transcripts per million (TPM) (expressed if TPM ≥ 0.5), and functional status (peptidase, pseudopeptidase, or uncertain) is inferred from analyses of conserved catalytic motifs.

A Tajima RR screen (FDR *q* ≤ 0.10) identified 12 *Hordeum* triplets with significant rate asymmetry (Figure 4; Table S9). Rate acceleration was concentrated in first + second codon positions (12/12 triplets) and was usually detectable when all positions were analyzed jointly (11/12), whereas third positions showed no significant asymmetry. A post‐hoc power check suggested that this absence is not explained by low information content alone (8/12 triplets would be expected to reach *α* = 0.05 at third positions if they had the same effect size as at first + second; binomial test, non‐significant), supporting an interpretation in terms of altered protein‐level constraints rather than a genome‐wide increase in mutation rate (Table S10). Moving to explicit codon models, RELAX showed no evidence of altered selection intensity in clade 13 (*K* = 1.12, LRT = 0.80, *p* = 0.371; *q* = 0.371) but detected intensified selection in clade 38 (*K* = 1.29, LRT = 5.40, *p* = 0.020; *q* = 0.040; BH across the two planned tests) (Figure 4; Table S11). Under RELAX, *K* > 1 indicates intensified selection, that is, a shift toward more extreme *ω* values relative to the background, which may reflect stronger purifying selection, stronger positive selection, or both. To test explicitly whether the focal clades also showed evidence of episodic diversifying selection, we then applied the gene‐wide BUSTED model. Gene‐wide BUSTED tests supported episodic diversifying selection in both focal clades (clade 38: nine foreground branches, LRT = 14.07, *p* = 4.39 × 10^−^^4^, *q* = 8.78 × 10^−^^4^; clade 13: 11 foreground branches, LRT = 9.02, *p* = 0.00550, *q* = 0.00550; BH across the two BUSTED tests; Table S12), indicating that *ω* > 1 occurred at a subset of sites on at least one foreground branch. Branch localization with aBSREL (multiple‐testing corrected) identified five branches with evidence of episodic *ω* > 1 (internal branches ID15, ID31, ID38, and ID40 and the terminal branch *H_chilense_*C1A_1; Table S13), consistent with brief bursts of positive selection on a small fraction of sites. At the site level, MEME detected 28 codon sites with evidence of episodic diversifying selection (*p* ≤ 0.10; Table S14), with 23 of these sites occurring within the *Hordeum* clade; mapping branch contributions yielded 43 independent substitution events across 26 *Hordeum* branches (Table S15). Using SLAC as a conservative baseline, we identified a small set of sites with pervasive selection signals (four sites under pervasive positive selection and 8 under pervasive purifying selection at *α* = 0.1; Table S16). These included the histidine residue of the catalytic site, which showed evidence of pervasive purifying selection. Grantham distances suggested that most of the non‐synonymous substitutions had a high impact on the protein's structure (22 cases), followed by moderate impact (12 cases) and low impact (nine cases).

Overall, the weak overlap between MEME‐positive sites and pervasively positive SLAC sites supports an evolutionary scenario dominated by episodic, lineage‐specific bouts of positive selection superimposed on a largely purifying/neutral background, alongside a subset of strongly constrained residues.

### 3.5 C1A homologs are largely expressed in *Hordeum* species and seem to display regulatory and functional specialization in different tissues

Comparative annotation of the *Hordeum* homologs in the InterPro database indicates that the gene encodes a papain‐like cysteine endopeptidase (PLCP) of the C1A peptidase family (Table S6) (Liu et al., 2018; Richau et al., 2012). Using presence/absence and sequence variation of the motifs typical of cystein proteases, we identified six homologs that have likely retained catalytic function: *H_comosum*_C1A_2 and *H_comosum*_C1A_3 (sequences are identical), *H_patagonicum*_C1A_1, *H_patagonicum*_C1A_2 (sequences are identical), *H_chilense*_C1A_4, and *H_stenostachys*_C1A_1 (Figure S4; Table S17). Other three homologs, *H_bogdanii*_C1A_8, *H_pubiflorum*_C1A_5, and *H_roshevitzii*_C1A_3, were classified as *“*uncertain,” as they contained substitutions in the Cys + WAFA 11‐amino acid window with unknown effect. The rest of the homologs are likely catalytically non‐functional (pseudopeptidases).

For proteins characterized based on sequence analysis as likely functional, uncertain, and likely nonfunctional, 3D models showed high local plDDT values (>90 in all residues of the catalytic triad and Gln24, Figure S5; Table S18), indicating a high degree of confidence in the given geometry. Across the functional groups, the Gln(Ser)24–Cys(Ser)30 distances ranged between 4.52 and 5.18 Å, Cys(Ser)30–His171(172) distances ranged between 3.49 and 3.74 Å, and His171(172)–Asn181(182) distances ranged between 4.86 and 5.04 Å. Across the three distances, the Kruskal–Wallis test indicated group differences for Gln–Cys(Ser) (*p* = 0.0129) and Cys(Ser)–His (*p* = 0.0199), but not for His–Asn (*p* = 0.0782). However, post hoc pairwise Mann–Whitney tests did not remain significant after BH correction. Medians suggested slightly shorter Gln(Ser)–Cys(Ser) (non‐functional 4.57 Å vs. functional 4.90 Å) and Cys(Ser)–His (3.52 Å vs. 3.62 Å) in non‐functional proteins, while His–Asn medians were comparable across classes. Overall, these analyses provide limited evidence against the null hypothesis: active‐site geometry appears broadly similar among classes, with only subtle shifts that did not survive multiple‐testing correction (Table S18). Overall, the models did not show any major deviations that would indicate nonfunctionality beyond the deviations we detected using the sequence analysis. Thus, we conclude that the proteins flagged as likely functional have retained catalytic activity, whereas likely non‐functional proteins lost the activity due to the loss or mutation of active sites. Proteins with *„*uncertain*‟* functionality have moderate changes in active sites, whose effect on function can not be assessed based on sequence analysis alone.

**FIGURE 5:**
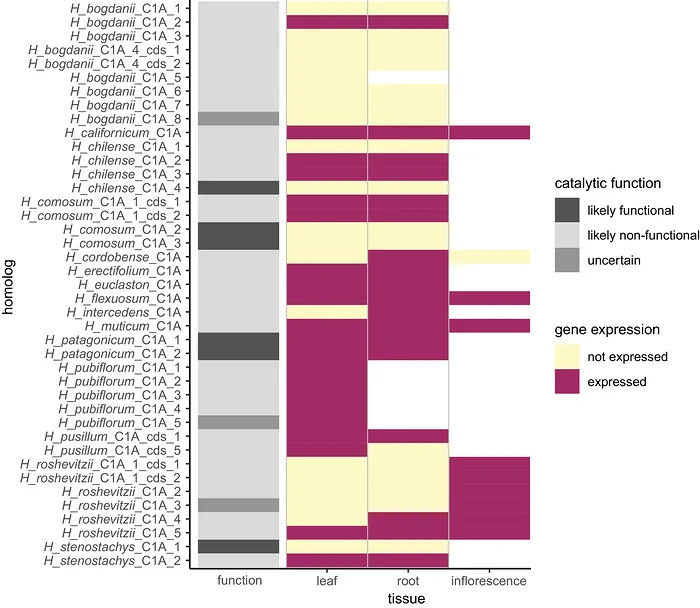
Expression pattern of C1A peptidase across *Hordeum* species and tissues. Rows correspond to individual homologs and columns to tissues (leaf, root, and inflorescence, where available). Homologs are additionally annotated by inferred catalytic functionality (likely functional, uncertain, likely non‐functional). A homolog was considered expressed if transcripts per million (TPM) was ≥0.5 and not expressed if TPM was <0.5.

We quantified gene expression levels across *Hordeum* species and tissues (primarily leaf and root, inflorescence in some species). Raw data are presented in Tables S19 and S20. On the log_2_(TPM + 1) scale, expression was readily detected in most species–tissue combinations, with clear interspecific, tissue‐specific, and homolog‐specific differences (Figure S6). Using TPM ≥ 0.5 as a presence threshold, most species retained multiple expressed homologs per tissue, with the number of expressed homologs varying markedly among tissues and species (Figure 5). Notably, *H. bogdanii* was the species showing the lowest proportional expression, with only one homolog showing TPM ≥ 0.5. Overall, in all *Hordeum* species, expression was detected in at least one homolog and one of the tissues measured. If we look at expression in relation to the potential functionality of the gene (likely functional, uncertain, or likely non‐functional), then expression was recorded in all three categories (Figure 5; Figure S6). In *H. roshevitzii*, a more detailed analysis involving six homologs/isoforms and seven different tissues showed expression of at least one homolog in all of the tissues, namely, in leaf, root, germinating grain, inflorescence, senescing leaf, caryopsis, and peduncle (Figure S7). This suggests a general (pleiotropic) role of the protein across organs, with tissue‐to‐tissue differences indicating regulatory and functional specialization.

## 4 DISCUSSION

### 4.1 The gene most likely originates from *Panicum*, but the specific donor is unclear

This study refines the evolutionary interpretation of a papain‐like cysteine peptidase locus previously proposed to be horizontally transferred into wild *Hordeum* (Mahelka et al., 2021). Across diploid *Hordeum*, the gene is broadly retained in sect. *Stenostachys* (absent only from *H. brevisubulatum* in our dataset), but it shows pronounced within‐genus dynamics. Copy number varies from one to eight paralogs per genome; locus length spans 327–13,885 nt largely due to transposable‐element insertions; and annotation yields numerous CDS isoforms (41 variants; 270–1275 nt). Importantly, C1A was probably not acquired as an isolated gene. Our previous BAC‐based study showed that in *H. bogdanii* and *H. pubiflorum* it is embedded in a *Panicum*‐derived chromosomal segment organized as tandemly repeated blocks of approximately 68 kbp, each containing several protein‐coding genes, including C1A peptidase (then referred to as Ervatamin‐C‐like) (Mahelka et al., 2021). Thus, at least part of the copy‐number expansion documented here is best interpreted as duplication/amplification of the larger HGT‐derived block rather than repeated tandem duplication of C1A itself. The whole‐genome assemblies analyzed here substantially improve confidence in C1A copy number across diploid *Hordeum* and further show that, once embedded in duplicated blocks, individual C1A copies followed partly independent evolutionary trajectories, including TE insertion, CDS diversification, and divergence in predicted function and expression. Together, these results indicate that after acquisition the transferred segment entered an active phase of block‐level amplification and subsequent copy‐specific remodeling, rather than persisting as a single, stable foreign insert. Although HGT was proposed as a likely mode of acquisition of the C1A peptidase in *Hordeum* (Mahelka et al., 2021), our recent work focusing on HGT at the genus level in *Hordeum* did not confirm this assumption (Feng et al., 2025): C1A peptidase, despite being a candidate, could not be confirmed as an unambiguous HGT because it did not pass filtering criteria—specifically, homologous sequences across Poaceae were insufficient for a robust phylogenetic analysis. In this study, we used a wide range of panicoid lineages to confirm that the C1A peptidase in *Hordeum* has its closest homologs in panicoid grasses.

In Panicoideae, close relatives of the *Hordeum* locus appear rare and patchily distributed. Among 77 phylogenetically verified accessions (48 species, 20 genera), PCR positives were confined to seven species of *Panicum* and *Paspalum*. When mapped onto a plastid *ndh*F tree, positives are scattered and polyphyletic, a pattern that is difficult to explain by strict vertical inheritance unless many independent losses are assumed (for Paniceae phylogeny, see Morrone et al., 2012).

The observed polyphyly within the *Panicum* and *Paspalum* clades could partly reflect polyploidy, reticulation, and ancient incomplete lineage sorting, all of which may contribute to discordance between plastid and nuclear phylogenies (e.g., Hunt et al., 2014; Scataglini et al., 2014). However, these processes alone are unlikely to explain the full distribution pattern, especially the occurrence of the C1A locus across more than one genus. A parsimonious explanation is that the relevant C1A lineage is genuinely uncommon in grasses and has experienced discontinuous gains by HGT and/or introgressive movement within Panicoideae.

Phylogenetic analyses place *Hordeum* homologs in a well‐supported clade with *Panicum* and *Paspalum* sequences and identify *Pan. bergii* as the closest sampled relative, refining earlier inferences based largely on *Panicum hallii* (Mahelka et al., 2021). The current South American distribution of *Pan. bergii*, nevertheless, contrasts with previous arguments for a Eurasian transfer (based on Eurasian members of sect. *Stenostachys* harboring the panicoid segment; Mahelka et al., 2017, 2021). Several nonexclusive scenarios could resolve this tension: (i) sparse taxon sampling may mean that *Pan. bergii* is a proxy for an unsampled Eurasian donor lineage within sect. *Panicum*; (ii) the donor lineage may have had a broader historical range than today; or (iii) the transfer occurred after *Hordeum* reached the Americas, followed by spread within the *Stenostachys* ancestor or via subsequent gene flow. In the past, *Hordeum* species hybridized, as evidenced by the existence of at least 19 allopolyploid species (Brassac & Blattner, 2015) and the proved exchange of chromosomal segments between different *Hordeum* lineages (Feng et al., 2025). Distinguishing these scenarios will require genomic‐context evidence (shared synteny and conserved flanking regions) rather than coding‐sequence similarity alone.

### 4.2 Duplication, TE remodeling, and selection contributed to divergence of C1A peptidase in *Hordeum*

The HGT‐derived block bears hallmarks of post‐transfer remodeling through duplication and TE activity (Feschotte, 2008; Lisch, 2013). TE insertions can promote rearrangements, alter local regulation and splicing, and accelerate pseudogenization, providing a plausible explanation for the observed range of gene sizes and CDS isoforms across *Hordeum* species. Although we did not examine these effects directly, TE insertions are known to affect gene function through changes in coding integrity, intron structure, splicing, and local regulatory context and may therefore have contributed to divergence among C1A copies. This structural volatility also creates an evolutionary testing ground: duplicated copies can be retained, lose catalytic function, or acquire new regulatory roles with reduced pleiotropic cost compared with a single‐copy gene (Conant & Wolfe, 2008; Force et al., 1999; Innan & Kondrashov, 2010; Ohno, 1970).

Sequence‐based tests indicate heterogeneous selective regimes among *Hordeum* copies. RR tests identified accelerated evolution on 12 *Hordeum* branches (*q* ≤ 0.10), with signal concentrated in first and second codon positions, consistent with shifts in amino‐acid constraint rather than a uniform rise in mutation rate. Branch‐site analyses further support episodic positive selection: aBSREL detected five positively selected branches after correction, and MEME detected 28 sites with evidence of episodic selection (*p* ≤ 0.10), many mapping to multiple independent events within *Hordeum*. For PLCP‐related proteins, such lineage‐specific bursts are plausibly driven by shifts in substrate preferences or interaction networks (including inhibitors), consistent with the lineage‐specific pattern reported here (Misas‐Villamil et al., 2016).

### 4.3 Peptidases and expressed pseudopeptidases coexist in *Hordeum* genomes

Motif‐based annotation suggests a mixture of catalytically competent peptidases and non‐peptidase homologs (pseudopeptidases). Only a minority of homologs retain the canonical C1A configuration (CGxC window, His and Asn of the catalytic triad, and the oxyanion‐hole Gln; Ménard et al., 1991; Storer & Ménard, 1994), whereas many show substitutions predicted to abolish proteolysis (e.g., Cys→Ser at the catalytic position) and a few carry atypical active‐site windows of uncertain effect. AlphaFold‐based models are consistent with these classifications: representative proteins generally fold into well‐resolved peptidase‐domain structures, and predicted active‐site geometry does not show gross disruption beyond the motif changes. Because PLCP activity also depends on maturation and cellular context (Liu et al., 2018; Richau et al., 2012; van der Hoorn et al., 2004), these predictions should be treated as hypotheses to prioritize functional assays (recombinant expression, activity tests, and inhibitor profiling). At this point, we cannot say with certainty whether a catalytically functional peptidase or a pseudopeptidase with an unknown function was introduced into the *Hordeum*. Unfortunately, the complete sequence of the gene in *Pan. bergii*, the closest related species, is unavailable, so it is impossible to assess whether the gene encodes a catalytically functional protein. Even if the complete sequence were available, it would be unclear whether the gene has retained its original function given that several million years have probably elapsed since its transfer to *Hordeum*, during which time it could have undergone functional divergence.

Gene expression data adds a key functional dimension. Transcripts are detected across *Hordeum* species and tissues, and expression occurs in all functional categories (likely functional, uncertain, and likely non‐functional). In *H. roshevitzii*, at least one paralog is expressed in each of seven tissues spanning vegetative, reproductive, and grain‐associated organs, indicating broad deployment rather than strict organ restriction, consistent with the broad, organ‐spanning expression reported for several PLCPs in other plant species (Liu et al., 2018; Richau et al., 2012). In this context, “expressed in all tissues” means that some paralog or isoform crosses the detection threshold in every sampled tissue, not that all copies are constitutively expressed at similar levels; the strong heterogeneity among paralogs and isoforms is more consistent with regulatory diversification than with redundancy (Conant & Wolfe, 2008; Force et al., 1999; Innan & Kondrashov, 2010; Richau et al., 2012).

Functionally, this diversification fits established PLCP biology. Active copies are compatible with roles in development and senescence, nitrogen remobilization, storage‐protein processing, and defense/stress responses (Liu et al., 2018; Richau et al., 2012). Meanwhile, expressed pseudopeptidases could contribute through non‐catalytic mechanisms—for example as binding or decoy proteins that sequester inhibitors or partners or as scaffolds that modulate protease networks—consistent with the broader view that catalytic loss does not preclude biological function (Eyers & Murphy, 2016; Reynolds & Fischer, 2015).

### 4.4 Horizontally transferred genes are dynamic components of eukaryotic genomes

Our results suggest that the *Hordeum* C1A locus is not merely a retained foreign gene, but part of a T‐DNA segment that has entered a phase of lineage‐specific diversification. The combination of block‐level amplification, TE‐associated restructuring, expression retention, coexistence of likely catalytic and non‐catalytic paralogs, and episodic positive selection indicates that this locus has become an evolving component of the recipient genome rather than a passive molecular relic. Horizontal transfer of a gene is only the starting point; once foreign DNA is integrated into the nuclear genome, it can follow multiple evolutionary trajectories. Many loci are rapidly lost or pseudogenized, whereas others persist long enough to be reshaped by local transposable‐element activity, duplication of the transferred insert, and progressive regulatory “domestication” into the recipient's gene networks (Mariault et al., 2025).

Work in grasses provides concrete examples of this continuum. In *Alloteropsis semialata*, dozens of horizontally acquired protein‐coding genes were detected in large genomic fragments (up to ∼170 kb) from multiple donors, and most are transcriptionally active (Dunning et al., 2019). More recent population‐genomic analyses within the same lineage inferred rapid gains and losses of horizontally acquired genes (roughly 6–28 acquisitions per million years, and losses of ∼11%–24% of gained genes per million years), with turnover far exceeding that of vertically inherited genes and generating an accessory gene pool that can fuel standing variation and, potentially, local adaptation (Raimondeau et al., 2023). At the family scale, a systematic scan of 17 grass genomes found HGT in most sampled species, with higher rates in rhizomatous taxa and among closer relatives, consistent with an interplay of opportunity and genomic compatibility (Hibdige et al., 2021). Functional co‐option after transfer is also documented, for example via horizontally acquired enzymes contributing to C4 photosynthetic biochemistry in *Alloteropsis* (Phansopa et al., 2020). From a temporal perspective, HGT can have both immediate and delayed adaptive effects. A delayed effect was observed in *Alloteropsis* grasses, where foreign genes that were initially selectively neutral hitchhiked with positively selected genes and subsequently contributed to local adaptation after the plants encountered a different environment (Olofsson et al., 2019).

Our results are consistent with a scenario in which the transferred C1A locus persisted long enough to enter a post‐transfer diversification phase, characterized by duplication, TE‐driven restructuring, transcriptional retention, and heterogeneous selection among copies. However, whether the ancestrally transferred copy was immediately adaptive upon arrival in *Hordeum* or instead initially represented standing variation that was later repurposed by selection remains unclear.

Comparable post‐transfer gene family expansions and divergence have been reported in other eukaryotes, supporting the idea that duplication and subsequent turnover can be a common “afterlife” of successful HGT. For example, fungal‐origin carotenoid biosynthesis genes in aphids expanded into multi‐copy families and diversified after an ancient transfer (Nováková & Moran, 2012). Similarly, fungal‐derived polygalacturonases in phytophagous beetles and mirid bugs show birth‐and‐death dynamics with lineage‐specific duplications, including both active enzymes and inactive paralogs, and these expansions have been linked to the evolution of herbivory and host‐range shifts (Kirsch et al., 2014; Xu et al., 2019). Horizontally acquired microbial cellulases in nematodes also show rapid copy‐number evolution and signatures of selection consistent with functional assimilation (Mayer et al., 2011). In plants, natural *Agrobacterium* T‐DNA insertions provide analogous examples where horizontally acquired sequences were duplicated and subsequently diverged within the host genome (e.g., duplicated cellular T‐DNA inserts in *Nicotiana otophora* and repeated/inverted IbT‐DNA copies in sweetpotato; K. Chen et al., 2018; Kyndt et al., 2015; Quispe‐Huamanquispe et al., 2019).

### 4.5 Conclusions and future prospects

In summary, our results support a panicoid origin of the *Hordeum* C1A locus, with *Panicum bergii* representing the closest sampled relative. The transferred locus appears to have persisted as part of a larger HGT‐derived DNA block that subsequently underwent duplication, TE‐driven remodeling, and functional diversification in *Hordeum*. Building on this foundation, future work that combines flanking‐region/synteny analyses with biochemical and genetic assays can refine the donor context and test which paralogs contribute catalytic versus non‐catalytic functions and whether they affect stress responses or development.

## AUTHOR CONTRIBUTIONS

**Václav Mahelka**: Conceptualization; funding acquisition; investigation; methodology; supervision; writing—original draft. **Petra Caklová**: Investigation; methodology. **Radim Čegan**: Investigation; methodology; writing—review and editing. **Simón Villanueva‐Corrales**: Investigation; methodology; writing—review and editing. **Jiřina Josefiová**: Investigation; methodology. **Jana Kneřová**: Investigation; methodology; writing—review and editing. **David Kopecký**: Conceptualization; funding acquisition; investigation; methodology; writing—review and editing. **Michaela Nagy‐Nejedlá**: Investigation; methodology. **Marek Szecówka**: Investigation; methodology; writing—review and editing. **Karol Krak**: Conceptualization; investigation; methodology; writing—review and editing.

## CONFLICT OF INTEREST STATEMENT

The authors declare no conflicts of interest.

## Supporting information



Supporting Information

Supporting Information

Supporting Information

Supporting Information

Supporting Information

## Data Availability

Multiple sequence alignments of *Hordeum* C1A peptidase are available in Supporting Information (Supporting Information Files S2, S3, S4), and chloroplast *ndh*F sequences of Panicoideae have been deposited in NCBI GenBank (for accession numbers see Table S8).
