# Supplementary material for: The afterlife of a horizontally transferred gene: Expansion and functional diversification of a C1A peptidase in wild Hordeum species
Source: Plant Genome. 2026 Aug 27;19(3):e70242. doi: 10.1002/tpg2.70242 (PMC13520124; doi:10.1002/tpg2.70242)

Supplementary material

The afterlife of a horizontally transferred gene: expansion and functional diversification of a C1A peptidase in wild *Hordeum* species

Václav Mahelka[1]*, Petra Caklová[2], Radim Čegan[3,4], Simón Villanueva Corrales[2], Jiřina Josefiová[2], Jana Kneřová[3], David Kopecký[3], Michaela Nagy-Nejedlá[2], Marek Szecówka[3], Karol Krak[1,5]

Supplementary file S1. Phylogenetic verification of Panicoideae accessions used for the PCR screening for the presence of C1A peptidase.

Phylogenetic verification of Panicoideae accessions used for the PCR screening for the presence of C1A peptidase

Taxon sampling

To assess the presence and sequence variation of the gene across Panicoideae, we screened a set of representative species. In our previous study, we showed that the gene occurs in diverse *Hordeum* species and *Panicum hallii*, but is absent from a broad set of other panicoid grasses, including additional *Panicum* species (Mahelka et al., 2021). When designing the Panicoideae screen, we also considered that most horizontally transferred genes in *Hordeum* appear to have donors in the genera *Panicum* and *Paspalum* (Feng et al., 2025). Therefore, our sampling was shallow across the subfamily Panicoideae—aiming to include at least one representative per subtribe sensu Morrone et al. (2012)—and dense within *Panicum* and *Paspalum* (Supplementary Table S2). Taxon names follow the NCBI Taxonomy Browser (https://www.ncbi.nlm.nih.gov/Taxonomy/Browser/wwwtax.cgi).

Plant material

Seeds were obtained from public seed banks (USDA, USA; IPK Gatersleben, Germany) or via inter-institutional seed exchange (Supplementary Table S2). Seeds were sown in growth chambers in 10 × 10 cm pots filled with a sand:seed-substrate mixture (2:1) and grown at 20 °C night / 30 °C day under an 8-h photoperiod. We used 0.1% KNO_3 in the initial watering. If seeds failed to germinate within two months, additional seeds were sown and kept under the same regime for another two months; only

0.01% gibberellic acid was added to stimulate germination. If seeds failed to germinate a second time, they were considered non-germinating. The non-germinating rate was 23%. In total, we successfully grew 91 samples representing 55 species from 23 genera (Supplementary Table S2). All specimens subsequently underwent taxonomic verification using a DNA barcode (see below).

Phylogenetic verification of Panicoideae accessions

Because many plants were weak and prone to mortality, we could not consistently grow them to the flowering stage for morphological identification. We therefore used DNA barcoding for taxonomic verification. We selected the chloroplast *ndh*F gene, for which a comprehensive phylogenetic framework integrating morphology is available (Morrone et al., 2012). A partial *ndh*F fragment (ca 800 bp) was amplified using primers 1318F (5′-GGATTAAC(CT)GCATTTTATATGTTTCG-3′) and 2110R (5′-CCCCCTA(CT)ATATTTGATACCTTCTCC-3′) (Olmstead & Sweere, 1994). PCRs used COMBI PPP mastermix (Top-BIO, Vestec, CZ) and 200 nM primers with the following cycling: 95 °C, 5 min; 35 cycles of 95 °C, 30 s – 50 °C, 30 s – 72 °C, 1 min; final extension 72 °C, 10 min; hold at 4 °C.

Amplicons were Sanger-sequenced from both strands and aligned to the *ndh*F dataset of Morrone et al. (2012). For species not published by Morrone et al. but present in our sampling, sequences were retrieved from NCBI and added (Supplementary Table S3).

To infer phylogenetic relationships, we ran maximum likelihood analyses on the IQ-TREE web server (Trifinopoulos et al., 2016) with *Bromus inermis* as the outgroup, automatic model selection, FreeRate heterogeneity, and 1,000 ultrafast bootstrap replicates. The primary dataset contained 332 samples (including the outgroup) aligned with MAFFT in Geneious. The multiple sequence alignment comprised 795 sites. Where multiple identical accessions existed for a species (e.g., Panicum capillare, Paspalum dilatatum), a single representative was kept and duplicates were removed from the analysis.

A taxon from our dataset was considered phylogenetically verified if:

(1) its sequence grouped with the same taxon (reference taxon) of Morrone et al. (2012) in the tree, or

(2) more than one accession of the same taxon was present in our dataset and these accessions formed a single clade, even if no reference taxon was available.

When a single accession lacked a reference taxon but occurred within the clade of the same genus (not necessarily monophyletic), it was flagged "unverified" but accepted.

Verification was rejected if:

(1) the tested accession did not group with the reference taxon (i.e., it fell in a different clade), or

(2) two or more accessions of the same taxon in our dataset occurred in different clades and no reference taxon was available.

Applying these criteria, 14 of 91 sequences were discarded (Supplementary Table S2). The remaining 77 accessions representing 48 species and 20 genera were used for PCR screening of the gene.

References

Feng J-W., Jiang, C., Strejčková, B. et al. (2025) A genus-wide pangenome of *Hordeum*. Research Square. doi: 10.21203/rs.3.rs-7766969/v1.

Mahelka, V., Krak, K., Fehrer, J., Caklová, P., Nagy Nejedlá, M., Čegan, R., Kopecký, D. and Šafář, J. (2021) A *Panicum*-derived chromosomal segment captured by *Hordeum* a few million years ago preserves a set of stress-related genes. Plant Journal 105, 5, 1141–1164.

Morrone, O., Aagesen, L., Scataglini, M.A., Salariato, D.L., Denham, S.S., Chemisquy, M.A., Sede, S.M., Giussani, L.M., Kellogg, E.A. and Zuloaga, F.O. (2012) Phylogeny of the *Paniceae* (Poaceae: Panicoideae): Integrating plastid DNA sequences and morphology into a new classification. Cladistics, 28, 333–356.

Olmstead, R.G. and Sweere, J.A. (1994) Combining data in phylogenetic systematics: an empirical approach using three molecular data sets in the Solanaceae. Systematic Biology, 43, 467–481.

Supplementary file S2. Multiple sequence alignment of C1A peptidase protein sequences from *Hordeum* and selected Panicoideae (broader sampling), used for phylogenetic analysis (step 1).

Supplementary file S3. Multiple sequence alignment of C1A peptidase protein sequences from *Hordeum* and selected Panicoideae (sampling restricted to clade A in Supplementary Fig. 2), used for phylogenetic analysis (step 2).

Supplementary file S4. Multiple sequence alignment of C1A peptidase coding nucleotide sequences (CDS) from *Hordeum* and selected Panicoideae (sampling restricted to clade A in Supplementary Fig. 2), used for phylogenetic analysis (step 3).

Supplementary Fig. S1. Gene tree of the C1A peptidase (CDS-based maximum-likelihood tree with transformed branch lengths) with branch IDs. The tree was pruned by removing *H. cordobense*.

Supplementary Fig. S2. Maximum-likelihood phylogeny of C1A peptidase homologs from *Hordeum* and their placement among Panicoideae, inferred from protein sequences. Blue indicates *Hordeum* homologs; magenta indicates PCR-amplified Panicoideae sequences generated in this study. Bootstrap support values <85% are not shown.

Supplementary Fig. S3. Maximum-likelihood phylogeny of C1A peptidase homologs from *Hordeum* and their placement among closely related Panicoideae, inferred from protein sequences. Blue indicates *Hordeum* homologs; magenta indicates PCR-amplified Panicoideae sequences generated in this study. Bootstrap support values <85% are not shown.

Supplementary Fig. S4. Conserved motifs of C1A peptidase homologs from *Hordeum*. Amino-acid numbering corresponds to alignment positions. Individual motifs are separated by five-residue gaps for clarity. The following motifs are highlighted: ERFNIN (59–78), GNFD (96–102), the oxyanion hole (182), the catalytic cysteine motif including WAFA (183–193), the catalytic histidine motif (229–239), and the catalytic asparagine (347). The histidine-site motif is shown in PROSITE pattern notation: residues in square brackets indicate allowed alternatives at a given position, residues in curly brackets indicate excluded residues, and {1,2} denotes a variable-length region of one or two residues.

Supplementary Fig. S5. Active-site geometry in C1A peptidase 3D models of *H_pubiflorum*_C1A_1 (A) and *H_patagonicum*_C1A_1 (B). Models comprise only the mature peptidase domain. Both models are shown in the same orientation and colored by pLDDT confidence (blue, high confidence, pLDDT ≥ 90; light blue/cyan, confident, pLDDT 70–90; yellow, low confidence, pLDDT 50–70). *H_pubiflorum*_C1A_1 (A) carries Ser30 at the nucleophile position and is therefore likely catalytically non-functional. *H_patagonicum*_C1A_1 (B) retains Cys30 together with His172 and Asn188 and is likely functional. The catalytic residues are highlighted (Gln24 – magenta, Ser/Cys30 – green, His171/172 – cyan, Asn187/188 – red), and inter-residue distances relevant to catalysis are indicated (Ser/Cys–His and His–Asn).

Supplementary Fig. S6. Expression heatmap of C1A homologs across *Hordeum* species and tissues. Rows correspond to individual homologs and columns to tissues (leaf, root, and inflorescence, where available). Homologs are additionally annotated by inferred catalytic functionality (likely functional, uncertain, likely non-functional). Color intensity represents transcript abundance on the log2(TPM + 1) scale.

Supplementary Fig. S7. Expression pattern of C1A peptidase homologs in *H. roshevitzii* across six homologs/isoforms and seven tissues. Rows represent individual homologs/isoforms and columns represent tissues. Homologs are additionally annotated by inferred catalytic functionality (likely non-functional, uncertain). (A) Binary expression status based on TPM, with homologs scored as expressed at TPM ≥ 0.5 and not expressed at TPM < 0.5. (B) Heat map of transcript abundance shown as log2(TPM + 1).

Supplementary Fig. S1

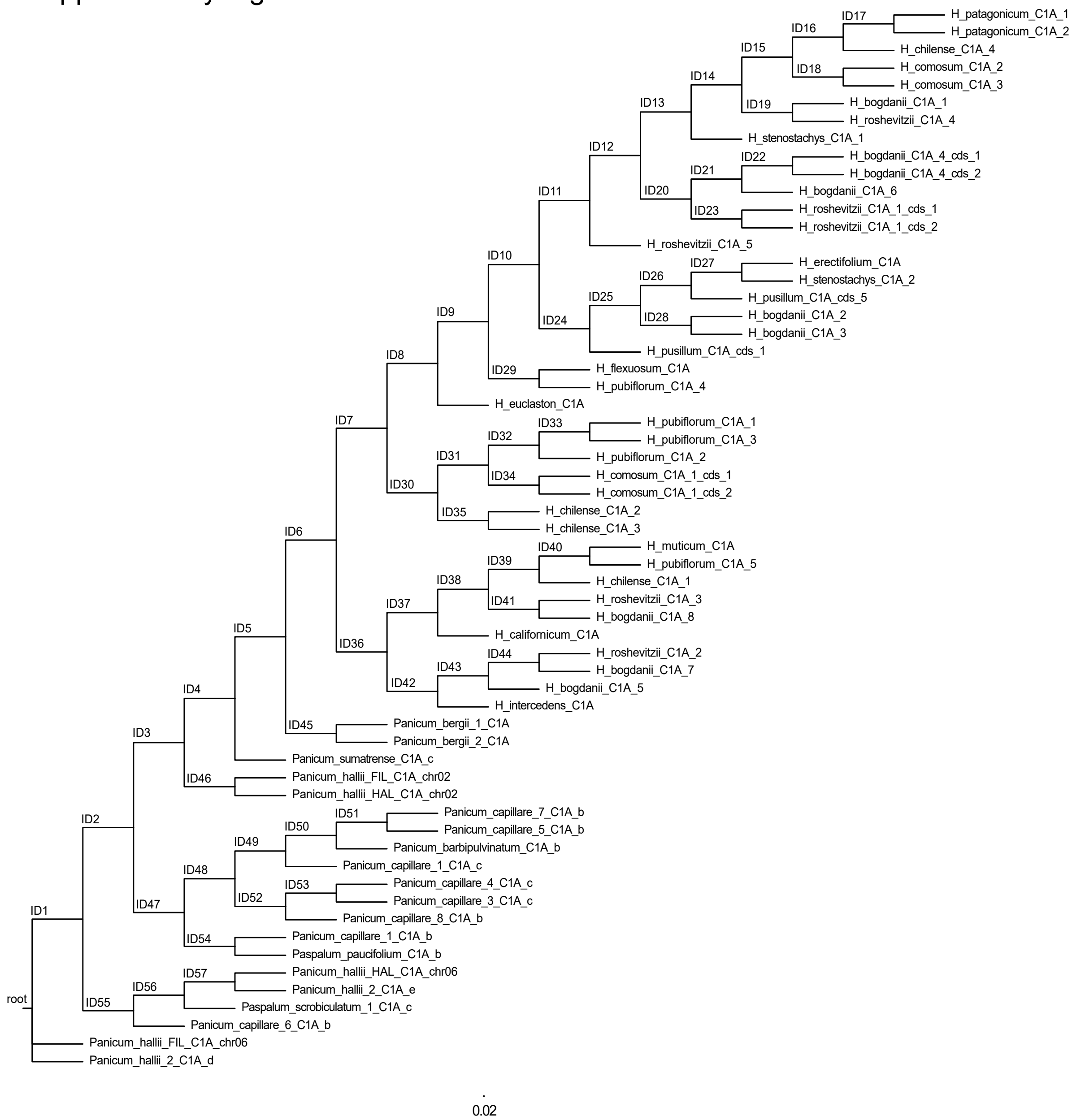

Supplementary Fig. S2

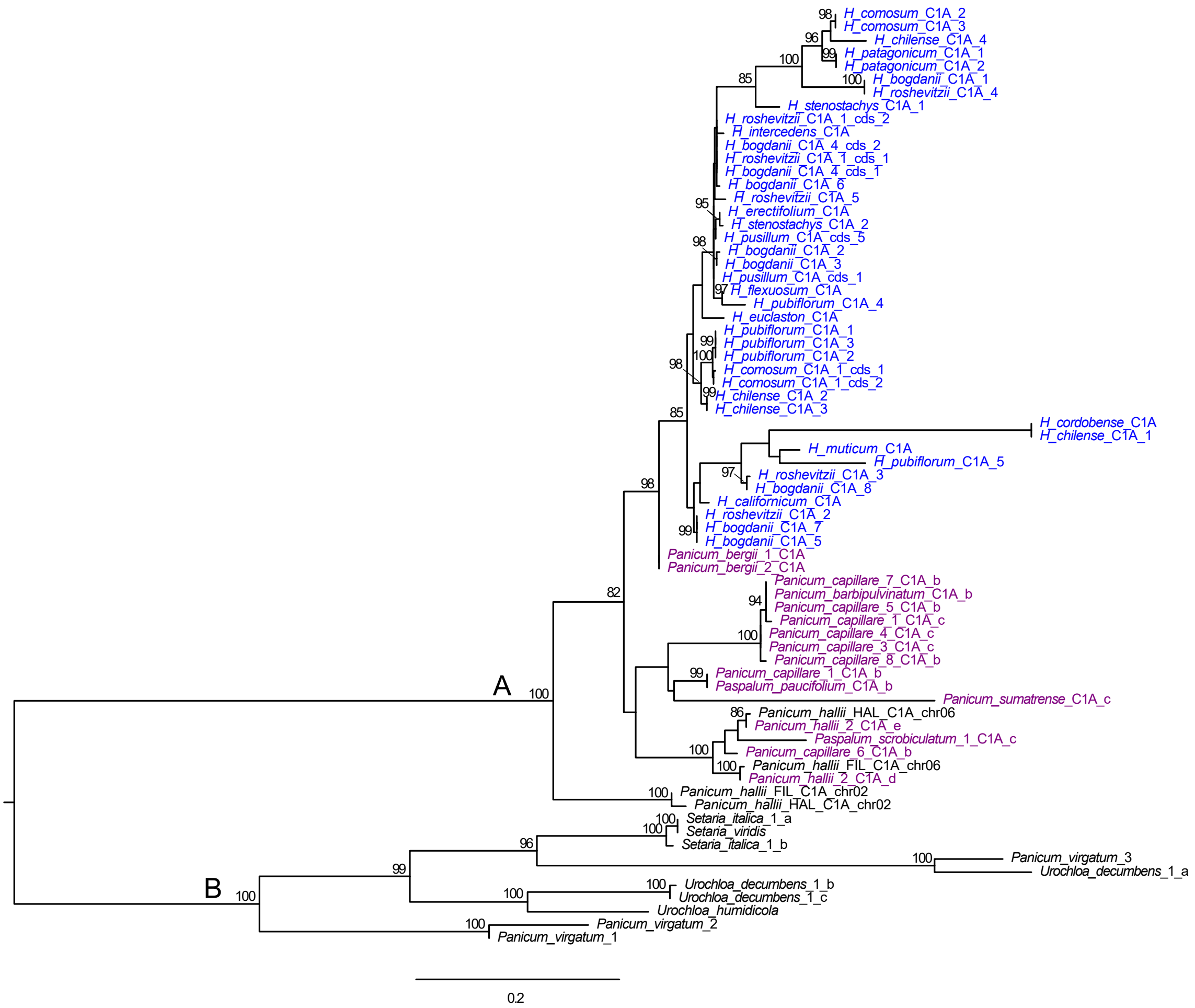

Supplementary Fig. S3

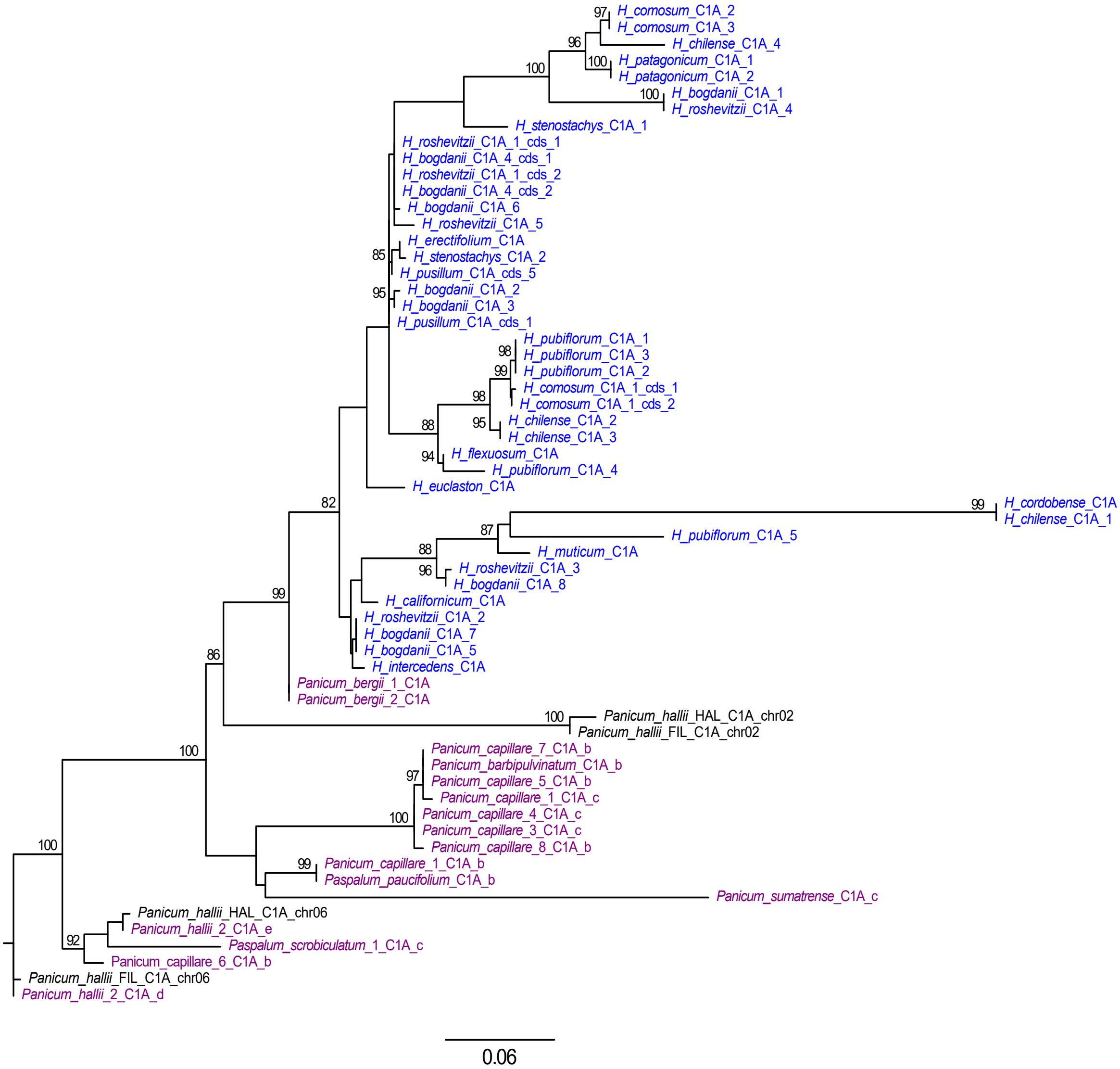

Supplementary Fig. S4

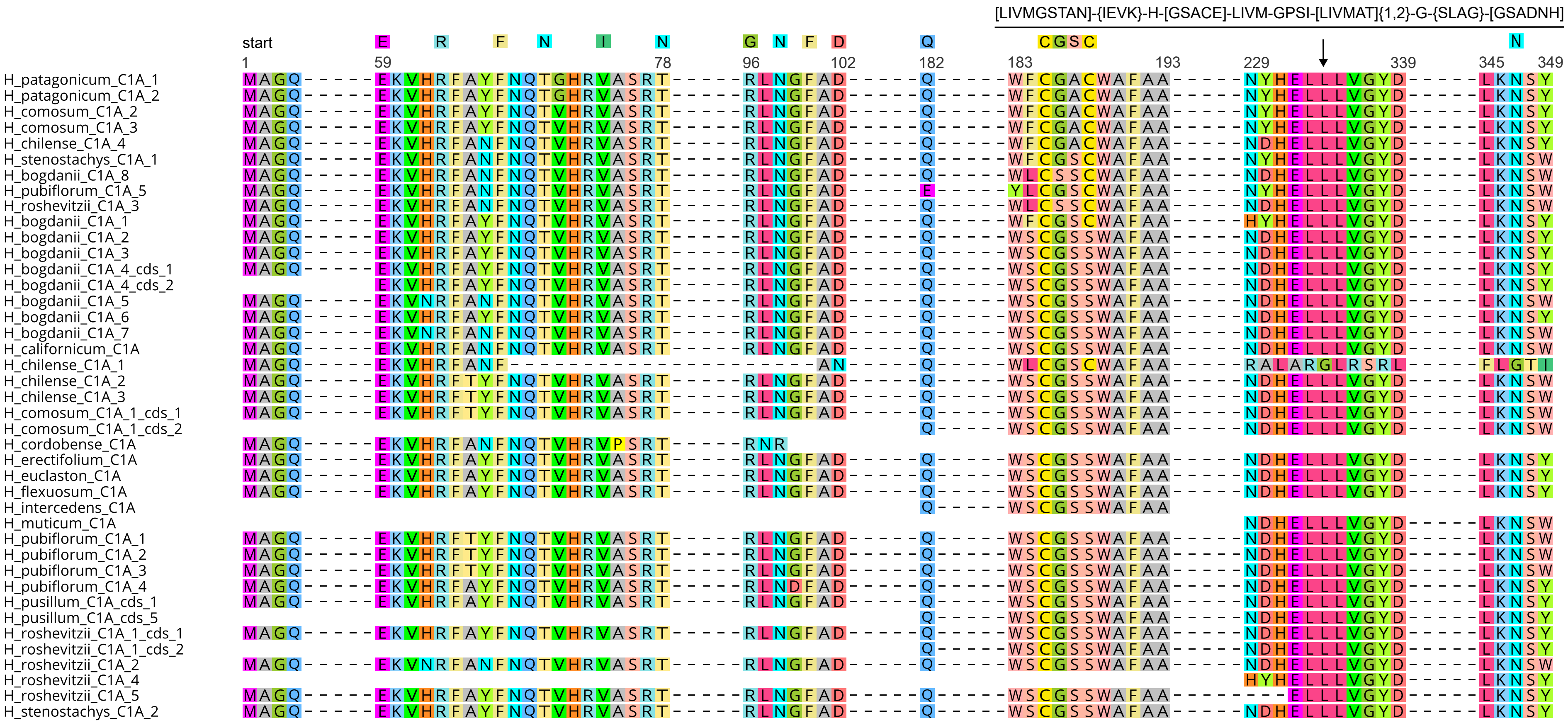

Supplementary Fig. S5

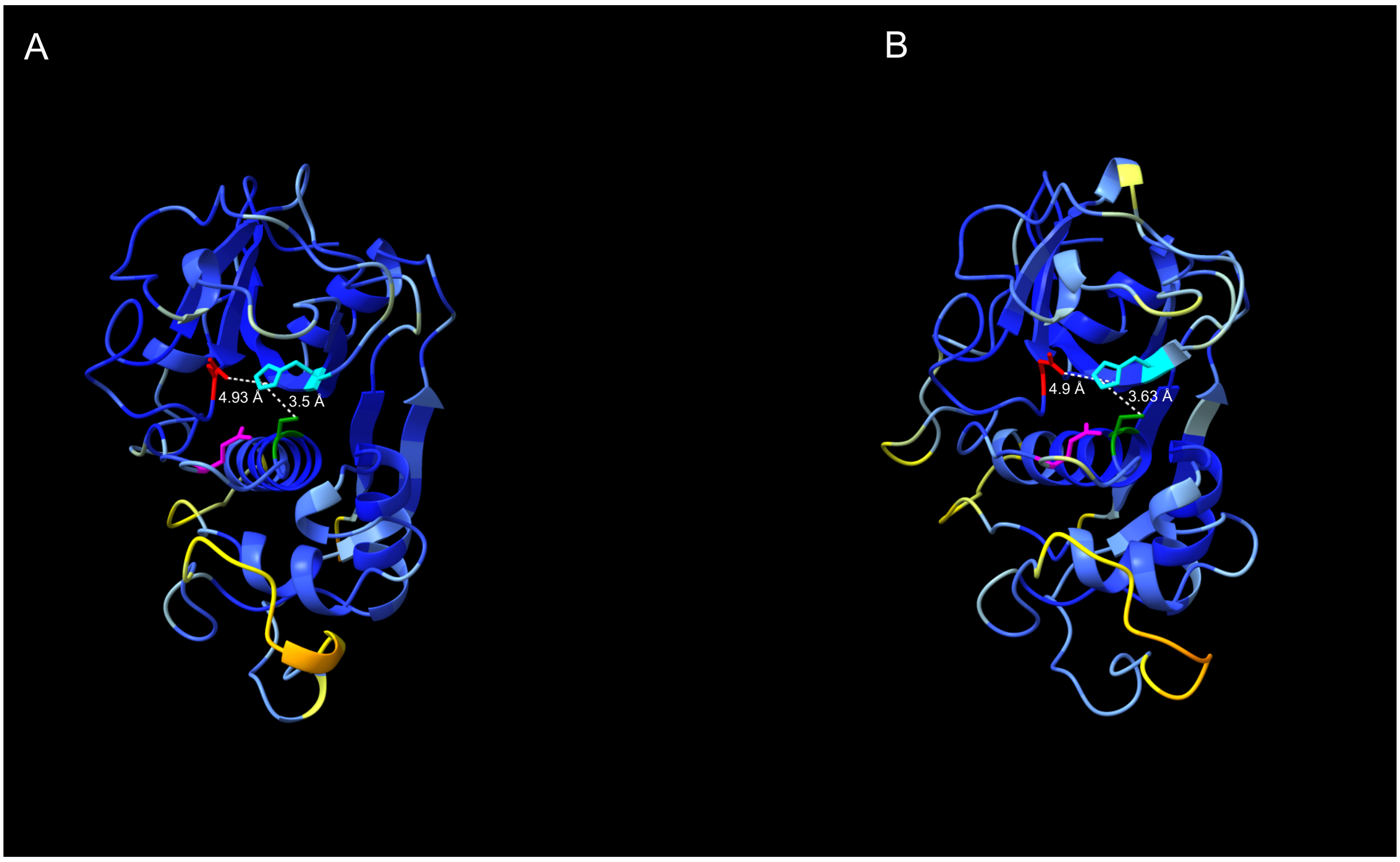

Supplementary Fig. S6

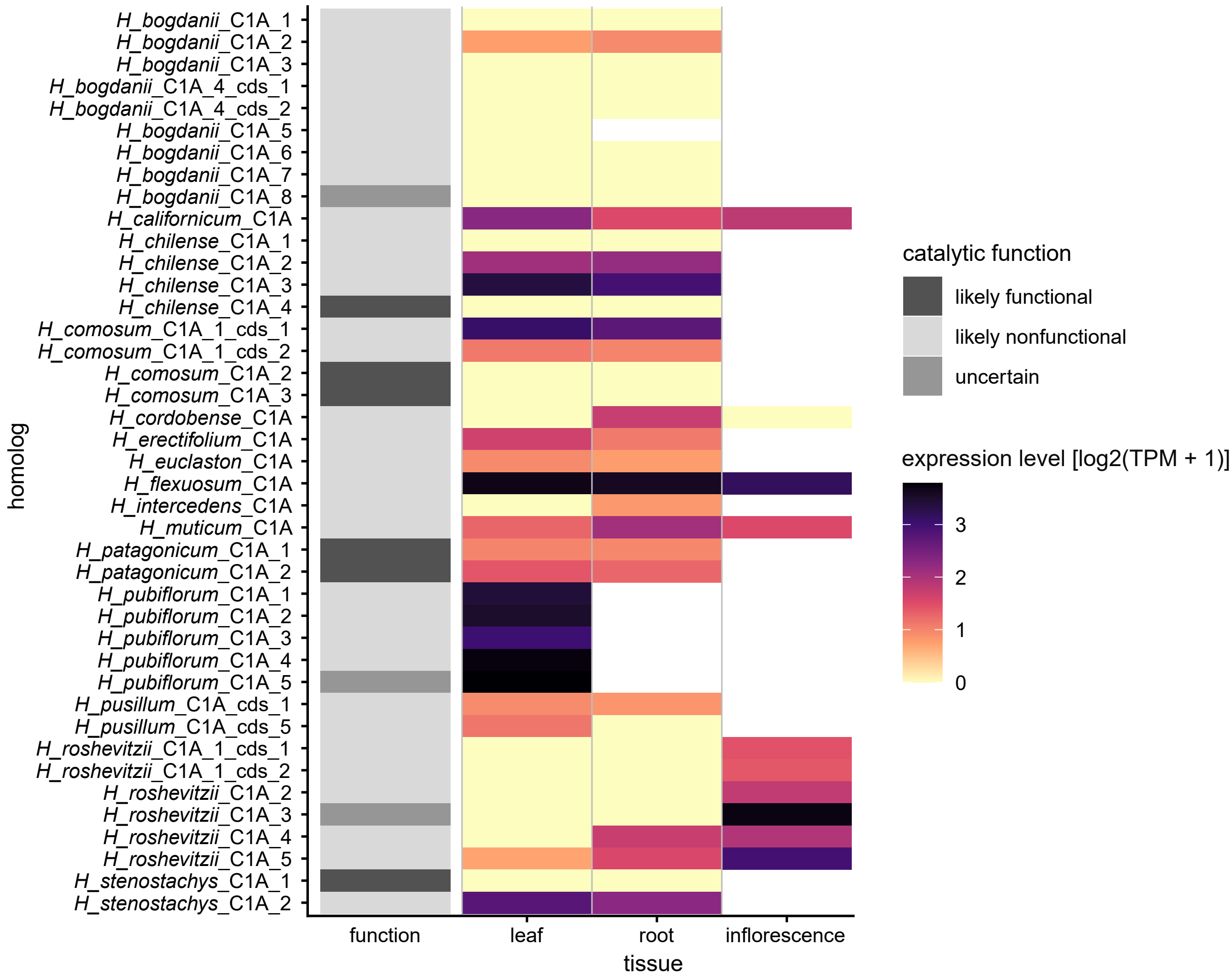

Supplementary Fig. S7

A

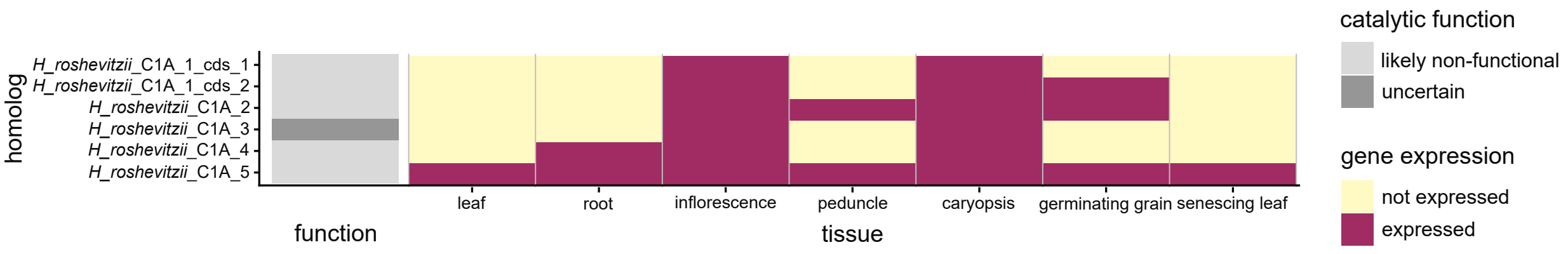

B

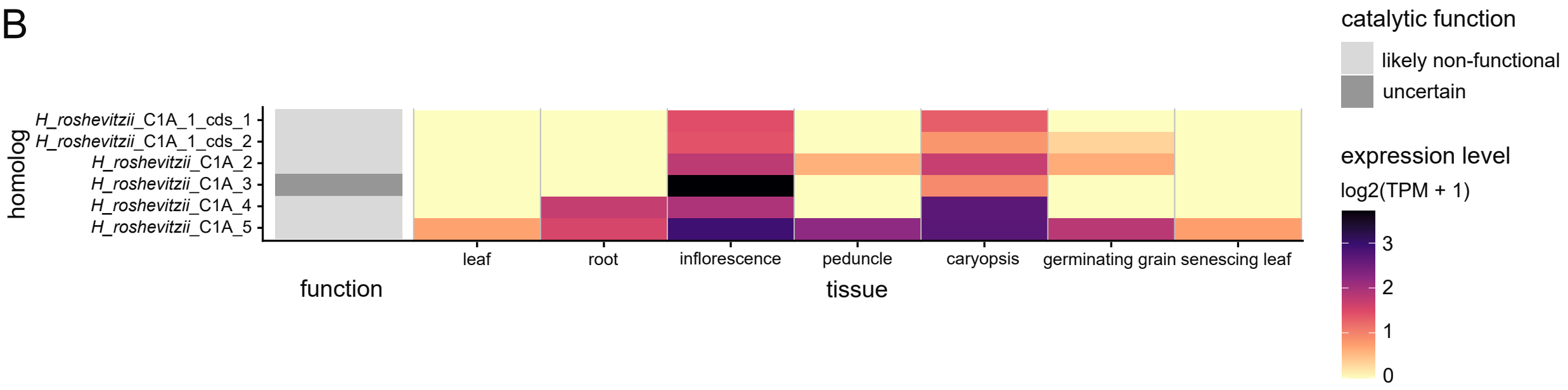

Supplement: Supplementary file 1 [file TPG2-19-e70242-s001.pdf]
